# Identifying Core Herbal Treatments for Children with Asthma: Implication from a Chinese Herbal Medicine Database in Taiwan

**DOI:** 10.1155/2013/125943

**Published:** 2013-08-28

**Authors:** Hsing-Yu Chen, Yi-Hsuan Lin, Peck-Foong Thien, Shih-Chieh Chang, Yu-Chun Chen, Su-Shun Lo, Sien-Hung Yang, Jiun-Liang Chen

**Affiliations:** ^1^Division of Chinese Internal Medicine, Center for Traditional Chinese Medicine, Chang Gung Memorial Hospital, No. 123, Dinghu Road, Gueishan, Taoyuan 33378, Taiwan; ^2^Graduate Institute of Clinical Medical Sciences, College of Medicine, Chang Gung University, No. 259, Wen Hwa 1st Road, Gueishan, Taoyuan 33302, Taiwan; ^3^Department of Medical Research and Education, National Yang-Ming University Hospital, No. 152, Xin Min Road, I-Lan 26042, Taiwan; ^4^Division of Chest Medicine, Department of Internal Medicine, National Yang-Ming University Hospital, No. 152, Xin Min Road, I-Lan 26042, Taiwan; ^5^Faculty of Medicine, School of Medicine, National Yang-Ming University, No. 155, Section 2, Linong Street, Taipei 112, Taiwan; ^6^Institute of Hospital and Health Care Administration, School of Medicine, National Yang-Ming University, No. 155, Section 2, Linong Street, Taipei 112, Taiwan; ^7^School of Traditional Chinese Medicine, College of Medicine, Chang Gung University, No. 259, Wen Hwa 1st Road, Gueishan, Taoyuan 33302, Taiwan; ^8^Institute of Traditional Medicine, School of Medicine, National Yang-Ming University, No. 155, Section 2, Linong Street, Taipei 112, Taiwan

## Abstract

Asthma is one of the most common allergic respiratory diseases around the world and places great burden on medical payment. Chinese herbal medicine (CHM) is commonly used for Taiwanese children to control diseases. The aim of this study is to analyze the CHM prescriptions for asthmatic children by using a nationwide clinical database. The National Health Insurance Research Database (NHIRD) was used to perform this study. Medical records from 1997 to 2009 with diagnosis with asthma made for children aged 6 to 18 were included into the analysis. Association rule mining and social network analysis were used to analyze the prevalence of single CHM and its combinations. Ma-Xing-Gan-Shi-Tang (MXGST) was the most commonly used herbal formula (HF) (20.2% of all prescriptions), followed by Xiao-Qing-Long-Tang (13.1%) and Xing-Su-San (12.8%). Zhe Bei Mu is the most frequently used single herb (SH) (14.6%), followed by Xing Ren (10.7%). MXGST was commonly used with Zhe Bei Mu (3.5%) and other single herbs capable of dispelling phlegm. Besides, MXGST was the core formula to relieve asthma. Further studies about efficacy and drug safety are needed for the CHM commonly used for asthma based on the result of this study.

## 1. Introduction

Asthma, characterized by a chronic inflammatory airway disease, is one of the most common allergic diseases among pediatric population around the world, affecting about 10%–20% of children in Europe and the United States and 11.9% in Taiwan [1, 2]. The high prevalence causes great burden on economics, about US$ 215 million reported in 2002 in Taiwan, and the health expense is still increasing yearly [3]. Currently, medical treatments are the main managements on asthma, such as steroid, beta-2 adrenergic agonist, leukotriene modifier, theophylline, and anti-IgE therapies, according to the guideline published by the Global Initiative for Asthma (GINA). However, the side effects of long-term use of steroid and beta-2 adrenergic agonist concerns parents a lot that growth, bone turnover, and adrenal gland function may be suppressed under higher dose of steroid [4, 5]. 

The use of complementary and alternative medicine (CAM) for diagnosis and treatment of pediatric diseases is not uncommon, especially for asthma [6, 7]. CAM includes treatments other than western medicine (WM), such as traditional Chinese medicine (TCM), kampo medicine, and homeopathy, by the definition of the National Center for Complementary and Alternative Medicine (NCCAM). In Taiwan, TCM is the most commonly used CAM among pediatric patients and widely used for respiratory disorders, allergic rhinitis, and gastrointestinal disorders [8]. Additionally, Chinese herbal medicine (CHM) is often used to treat diseases when compared with other TCM modalities, for example, acupuncture, massage, or moxibustion [9]. 

Nevertheless, the evidence of CHM used for asthma is still insufficient; only small amount of CHM was proved by randomized clinical trials [10]. The role of the CHM with proven effectiveness on treating asthmatic children remains unclear, and, on the other hand, the prescription patterns of CHM used for asthma are not known currently. Without this information, recommendations on clinical practice cannot be made, and further clinical trials are unable to be done to judge the efficacy of the CHM commonly prescribed by TCM doctors. The high complexity of CHM prescription may be the main cause for this problem. In average, four to five CHM are usually used in one prescription, and about 700 CHM are available in Taiwan for TCM physicians [11–13]. Additionally, the combinations of CHM should comply with the TCM rules and mainly depend on characteristics of CHM and patients' TCM syndrome. The specific combination patterns reflect TCM's viewpoints on diseases' nature and managements. Also, the core formula, which is defined as a CHM commonly used and combined with others, is the center of all prescriptions on treating a specific disease, and it can only be revealed after analyzing the combinations patterns of CHM [12]. Prescription analysis of CHM prescription is the key to understand the commonly used CHM and the CHM combination patterns for a disease. 

The aim of this study is to analyze the CHM prescriptions used for asthma among children in Taiwan. Based on these epidemiological statistics, more attention could be focused on efficacy and safety of the CHM which is used more commonly, especially the core formula. 

## 2. Materials and Methods

### 2.1. Data Source

The National Health Insurance Research Database (NHIRD) was used to perform CHM prescription analysis in this study. The NHIRD was composed of every medical record reimbursed by the National Health Insurance (NHI) in Taiwan. The NHI in Taiwan had two unique features: high coverage of insured populations and total reimbursement of the usage of TCM. To date, Taiwan is the only country around the world which fully paid the medical expense of TCM, including Chinese herbal medicine, acupuncture, massage, and moxibustion. Therefore, people in Taiwan may freely choose western medicine (WM) or TCM treatments for diseases without bias. Additionally, from 1995, the year the NHI started, to the present day, the coverage of the NHI increased yearly and approached 100% of total populations. The high coverage of the NHI makes the NHIRD become a nationwide database since nearly all medical records of Taiwanese were included in this database. This database can be applied and analyzed by researchers as long as the study protocol is approved by the Institution Review Board (IRB). As a nationwide database, the NHIRD has been successfully used for numerous studies, including cohort or cross-sectional design, about WM or TCM utilization status in Taiwan [12, 14, 15]. 

Detailed clinical information of every medical record from ambulatory visits and hospitalization was included in the NHIRD, and thus it was feasible to perform large-scale statistical analysis on prescription among pediatric asthmatic patients. For ambulatory visits, contents of the NHIRD contained patients' identification number, gender, birthday, visit date, diagnosis, faculty, doctor, management, and prescription, in which the dose, frequency, and duration of medication were provided. The International Classification of Diagnosis, 9th revision, with Clinical Modification (ICD-9-CM) was used to represent diagnosis of every visit, and three diagnoses could be used for a visit at most. Furthermore, the first diagnosis was requested to be the major reason for a visit. 

### 2.2. Selection of Study Subjects

**Figure 1:**
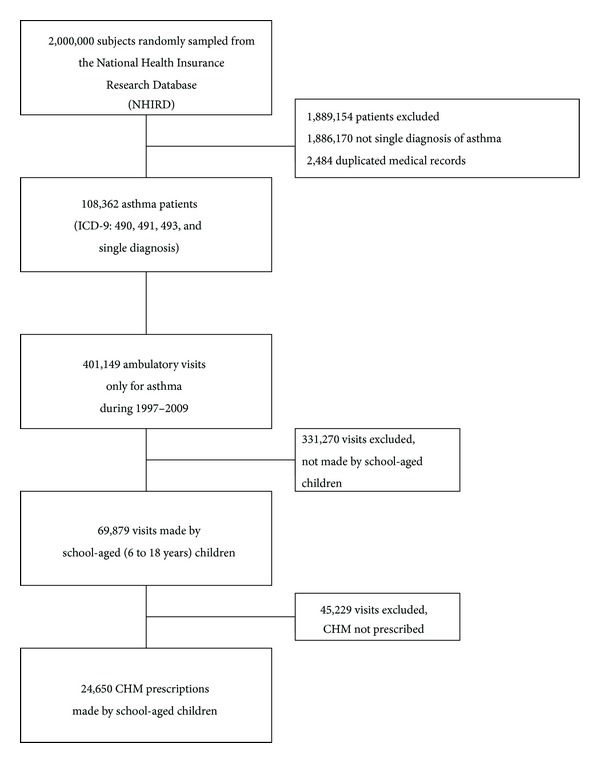
Flow diagram of enrollment of subjects.

To perform the prescription analysis on pediatric TCM users with asthma, a composite two million people sample dataset was used, and the prescriptions made by these patients during January 1, 1997, and December 31, 2009, were extracted from the whole NHIRD. The sample process was done randomly by the Bureau of National Health Insurance, the administration of the NHIRD. No differences of patients' gender and age was found between sample and whole datasets; thus, the sample dataset could be used as the representative of general population. To identify the pediatric TCM users for asthma, a series process was used and illustrated in Figure 1. ICD-9 of 490–493.x, except 492.x, was used to identify asthmatic patients. Only visits with single diagnosis of asthma were included into final analyses in order to eliminate the possible confounding factors caused by the comorbidities.

Additionally, age restriction of six to eighteen years was used to acquire asthmatic children. It was reported that wheezing and low lung function in early childhood may be related to small airway but not asthma, and therefore, the pathogenesis may be quite different from the asthma diagnosed in later childhood [16]. To reduce the confounding factors of prescription due to different pathogenesis, the lowest age restriction was set to six years. Meanwhile, the upper limit was set to eighteen years in order to focus the study population on children. 

Furthermore, only ambulatory visits with CHM prescriptions were included into the final analysis. Visits with acupuncture, massage, or moxibustion were excluded to avoid the potential influence on prescriptions. 

### 2.3. Study Variables

Frequencies, durations, and dosages of commonly used CHM and its combinations were used to demonstrate the prescription patterns for asthmatic children. Two kinds of CHM were reimbursed by the NHI in Taiwan: single herb (SH) and herbal formula (HF). HF were the mixture of multiple SH in fixed proportion, and the proportion must be the same as the HF records in TCM classics. HF and SH were all made into concentrated powder by the Good Manufacturing Practice pharmaceutical factories. Additionally, the indication, composition, and TCM properties, such as four qi and five flavors, were provided by the Committee on Chinese Medicine and Pharmacy, the administration of CHM in Taiwan. The characteristics and role of HF or SH among prescriptions could be disclosed by linking the corresponding CHM information to the prescriptions in the NHIRD.

### 2.4. Statistical Analysis

Descriptive statistics were used to present the frequencies of the most commonly used CHM, and association rule mining (ARM) was used to analyze the combinations of CHM for asthmatic children. ARM was one of the data mining techniques, and it was developed to explore significant combinations among huge database. While the amount of clinical data increased rapidly, ARM was successfully applied into various kinds of studies in both in WM and TCM categories, such as the analysis on the comorbidities of a disease and prescription analysis of CHM on diseases [11, 12, 17, 18]. Support and confidence factors were two key factors in ARM to determine the significance of combinations. Support factors were the prevalence of every CHM among all prescriptions, and this factor was used at first to filter out the uncommon CHM. Second, confidence factor was used to determine the strength of connections between every two CHM in combinations. The confidence factor was similar to the concept of conditional probability, and it could be presented as probability (CHM A *∩* CHM B)/probability (CHM A) while both CHM A and CHM B were prescribed commonly. Only the combinations that passed the examination of confidence factor were thought to be significant. The threshold value of support and confidence came from the consensus between all coauthors and the experiences from previous works. For the analysis on the prescription patterns of asthmatic children, support factor was set to 1% and confidence factor was set to 20%, and the free software “R” (version 2.15) with “arule” model was used.

Furthermore, the network of CHM commonly used can be established by illustrating the significant combinations. The core formula can be disclosed on the center of all CHM, and the freeware NodeXL (http://nodexl.codeplex.com/) was used to express the network of CHM for pediatric asthma. 

### 2.5. Ethical Consideration

The identification numbers of all subjects in the NHIRD were well encrypted, and therefore it was impossible to trace any private information. The protocol of this study was closely examined by the institutional review board of the Chang Gung Memorial Foundation (IRB no.: 101-3032B).

## 3. Results

**Figure 2:**
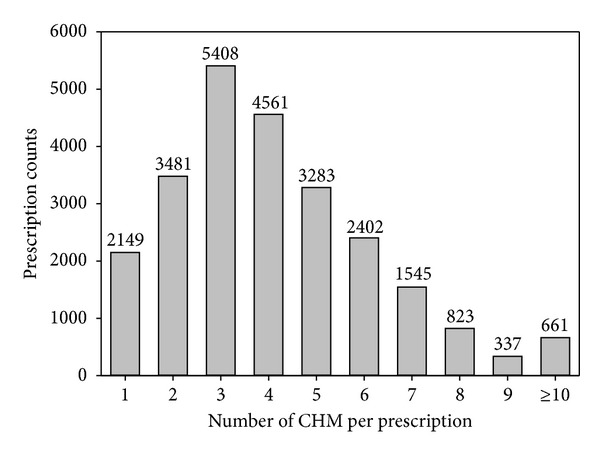
Distribution of numbers of CHM per prescription.

A total of 108,362 patients had diagnosis of asthma at least once during 1997–2009, and a total of 401,149 ambulatory visits were made. 69,879 visits were made by the children aged from six to eighteen. Additionally, a total of 24,650 CHM prescriptions could be extracted for further analysis (Figure 1). In average, each prescription was composed of 4.2 CHM, and TCM physicians usually used up to three to four CHM in one prescription (Figure 2). This result reflected that multiple CHM in one prescription was a common phenomenon, and this was compatible with similar works on other diseases [12].

### 3.1. The Most Commonly Used Single Herbal Formula (HF) and Single Herb (SH)

Ma-Xing-Gan-Shi-Tang (MXGST) was the most commonly used HF for asthmatic children (20.2%), followed by Xiao-Qing-Long-Tang (XQLT) (13.1%), Xing-Su-San (12.8%), Mai-Men-Dong-Tang (10.7%), and Zhi-Sou-San (10.5%) (Table 1). On TCM's viewpoints, lung was the primary organ involved in asthma, and pathogens with exterior heat or cold may precipitate asthma.

Additionally, Zhe Bei Mu was the most frequently used SH, and it could be found in around 15% of prescriptions. The markedly high prevalence of Zhe Bei Mu, even higher than most HF, symbolized its significance for asthma. As the second and third commonly used SH, both Xing Ren and Jie Geng had around 10% prevalence, followed by the Huang Qin (8.1%) and Yu Xing Cao (6.6%) (Table 2). Moreover, the indication for SH concentrated on resolving phlegm in lung and, more interestingly, relieving constipation. Furthermore, the daily dose of SH was usually less than HF, about one-third to one-fourth, and the differences in dose revealed the adjuvant role of SH in contrast to HF.

### 3.2. Combinations of Two CHM Commonly Used for Asthma

**Figure 3:**
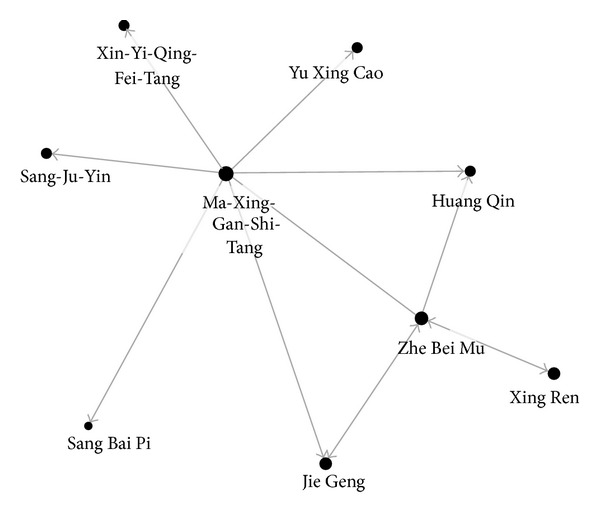
Network of CHM combinations for asthmatic children.

Ma-Xing-Gan-Shi-Tang with Zhe Bei Mu was the most commonly used two CHM in combination (3.5%) (Table 3). Furthermore, the network of CHM could be illustrated as in Figure 3, and the central role of Ma-Xing-Gan-Shi-Tang can be clearly shown. Ma-Xing-Gan-Shi-Tang had the highest prevalence and the most connections with other CHM. Subsequently, Ma-Xing-Gan-Shi-Tang was the core formula for asthmatic children. Furthermore, only Ma-Xing-Gan-Shi-Tang was frequently used to combine with other CHM. This fact disclosed the importance of analyzing combinations of CHM in addition to single CHM since most prescriptions were composed of several CHM.

### 3.3. External Validation of CHM Effects

To explore possible pharmacological mechanisms of CHM as external validation, extensive literature search was done in PubMed (last assess on April 20, 2013). Search keywords were composed of every CHM name and asthma-related words, such as bronchitis, allergic rhinitis, allergy, and hyper responsive airway. CHM names in English, Latin, and Chinese (pin yin) were used as keywords when searching references, and, additionally, the corresponding names in Kampo medicine and Korean medicine were included in searching words to avoid omission. The immunomodulation effects on suppressing type 2 T-helper cells and decreasing subsequent cytokine secretion were exclusively found in nearly all commonly used CHM; however, only HF had bronchodilation effect (Table 4).

## 4. Discussion

The key finding of this study is the recognition of MXGST as the core formula for treating asthmatic children. To the best of our knowledge, this is the first study to demonstrate the prevalence and the prescription patterns of CHM prescribed for asthmatic children. Also, the combination patterns were discovered by applying ARM to the nationwide prescription database. Further careful examination for efficacy and safety of MXGST should be carried out since the prevalence is relatively high among all available CHM, and this result from a nationwide study could be regarded as a form of consensus from the majorities of TCM physicians. MXGST is one of the most famous formulas, and composition and indication were shown in the ancient TCM classic, “Shanghanlun”. MXGST has been widely used for infectious and inflammatory airway diseases for thousands of years. The indication for MXGST includes wheezing, cough, coarse breathing sound, thirst, and fever; the hot-type asthma in TCM terminology [19]. 

The high prevalence of MXGST raises demand on evidence of efficacy which is still lacking. The effects on asthmatic children may be related to beta-2 adrenoreceptor agonist with subsequent bronchodilation and antitussive effect [20, 21]. Moreover, the anti-influenza effects of MXGST may be another reason for asthma since respiratory tract infection is reported to be an important trigger for asthma [22, 23]. Ma Huang, the most important part of four ingredients of MXGST, is extensively used as a bronchodilator by TCM physicians for a long time, such as Xiao-Qing-Long-Tang (rank 5 in HF, 10.5% of all prescriptions) and Ding-Chuan-Tang (rank 6 in HF, 9.8% of all prescriptions, not shown in table) [24–26]. Ephedrine and pseudoephedrine contained in Ma Huang are the main ingredients for brochodilation, and by combining them with Gypsum Fibrosum in MXGST, the unwanted physiological responses of Ma Huang may be balanced, such as hyperthermia and body fluid reduction [27].

In addition to use alone, MXGST was usually used with other CHM, especially SH, and phlegm was the most common indication of SH (Table 3). Phlegm, on TCM's viewpoint, is the same as sputum and is pathologic secretions from allergic airway. Airway secretion is a crucial factor of mortality and morbidity caused by asthma, and it is more important among children [28, 29]. Goblet cell and submucosal gland stimulated by Th2-related cytokine and activation of inflammation, such as mitogen-activated protein kinase-related pathway, are the main causes of airway hypersecretion [30]. Inhibition of inflammatory pathway and decreasing Th2-related response can be achieved by phlegm-dispelling SH, for example, Zhe Be Mu, Xing Ren, Jie Geng, Huang Qin, and Yu Xing Cao. Therefore, adding these SH with MXGST enhances the relative weak effect of MXGST on decreasing airway secretion and avoids possible overdose of MXGST. 

In contrast to synergistic effects, the SH commonly used to combine with other SH or HF raises the importance of safety issues, for example, food allergy from Xing Ren (*Prunus armeniaca* L.) and Yu Xing Cao (*Houttuynia cordata* Thunb.) Both SH were reported to possibly induce severe allergic reactions. Injection of Yu Xing Cao was not recommended for children due to close connection to anaphylactic shock [31]. Though only oral form of Yu Xing Cao is approved to market in Taiwan and dose was much lower than injection form, it is still urgent for TCM doctors to put more emphasis on this issue. In addition, Pru p 3 is a lipid transfer protein abundant in Xing Ren which is also a major allergen for IgE activation. Although Pru p 3 can be unfolded when boiling, which is the routine procedure for preparation of Xing Ren, and reduces the antigenicity [32, 33], the awareness of potential allergic reaction should be kept by TCM physicians. 

Furthermore, different triggers for asthma attack among children may influence the prescription of CHM. Xiao-Qing-Long-Tang (XQLT) is the second most commonly used HF for asthmatic children, and it has extensive effects on decreasing Th2-related immune response, anti-inflammatory, reducing secretion, and has potent bronchodilator effect [25, 26, 34–36]. Also, it is effective in controlling allergic rhinitis [37]. Asthma triggered by cold exposure and weather change was the most common indication for XQLT, and it is quite different from asthma triggered by respiratory infection, which is the most important indication for MXGST. Children constitution is the main cause of different triggers to the same disease. In Taiwan, asthma triggered by respiratory tract infection is much more common than that triggered by cold exposure and weather change among asthmatic children. The different prevalence in triggers of asthma adequately reflects the prescription patterns of CHM.

The major advantage of this study is to explore core formula and common CHM combinations for asthmatic children among a nationwide clinical database. ARM is a powerful tool to find out the important CHM combinations among large number of prescriptions, and the combination patterns are an important reference for TCM doctors because TCM doctors usually use multiple CHM in one prescription when treating diseases. Also, the commonly used CHM is not necessarily the important CHM used in combination, and therefore, the study of combination patterns is important when analyzing CHM prescriptions; for example, XQLT was not found in commonly used two CHM in combination although it was the second most HF used for asthma (Tables 2 and 4). 

However, there are still some limitations for this study. First, the number of CHM prescriptions for final analysis may be substantially reduced since the single diagnosis of asthma is used. However, this procedure may promptly reduce the influence on prescription from comorbidities, such as allergic rhinitis. Since allergic rhinitis highly coexisted with asthma, the use of single diagnosis was helpful in focusing on prescriptions for asthma. Second, the folk medicine in Taiwan was not included in the analysis in this study since folk medicine was not reimbursed by the NHI. Only CHM that is produced by the GMP factory and that passed the examination of the CCMP is approved to be prescribed, and thus, the result of this study is readily used for further studies. Third, CHM with higher prevalence could not directly correspond to better effectiveness. The precise efficacy of CHM should be evaluated carefully by conducting well-designed randomized, placebo-controlled, and double-blinded clinical trials. The CHM explored in this work would be valuable references to the choice of CHM for clinical trials.

## 5. Conclusion

MXGST was the core formula for asthmatic children, and it was commonly used with phlegm-dispelling SH. By analyzing the nationwide clinically relevant prescription database, this result may be regarded as a form of consensus from TCM physicians in Taiwan. Still, further stringently designed clinically relevant randomized clinical trials for efficacy and safety study about the commonly used herbal preparations in the treatment of asthma discovered in this study are warranted.

## Figures and Tables

**Table 1:** Herbal formulae (HF) frequently used for asthmatic children in Taiwan (total prescriptions: 24,650).

Latin name	Composition	Indication	Average dose (gm/day)	Prevalence (%)
Ma-Xing-Gan-Shi-Tang (MXGST)	Ma Huang (*Ephedra sinica *Stapf, *Ephedra intermedia* Schrenk et C. A. Mey, or *Ephedra equisetina* Bhe.), Xing Ren (*Prunus armeniaca* L. var. *ansu* Maxim.), Shi Gao (Gypsum Fibrosum), and Gan Cao (*Glycyrrhiza uralensis* Fisch.)	Heat evils congest the lung	14.7	4,986 (20.2%)

Xiao-Qing-Long-Tang (XQLT)	Ma Huang (*Ephedra sinica *Stapf, *Ephedra intermedia* Schrenk et C. A. Mey, or *Ephedra equisetina* Bhe.), Gui Zhi (*Cinnamomum cassia* Blume), Ban Xia (*Pinellia ternata* (Thunb.) Breit), Gan Jiang (*Zingiber officinale* Rosc.), Xi Xin (*Asarum heterotropoides* F Schum. var. *mandshuricum* (Maxim.) Kitag., or *Asarum sieboldii* Miq), Wu Wei Zi (*Schisandra chinensis* (Turcz.) Baill), Bai Shao Yao (*Paeonia lactiflora* Pall.), and Gan Cao (*Glycyrrhiza uralensis* Fisch.)	Exterior wind-cold with internal accumulation of retained fluid	13.5	3,225 (13.1%)

Xing-Su-San	Xing Ren (*Prunus armeniaca* L. var. *ansu* Maxim.), Zi Su Ye (*Perilla frutescens* (L.) Britt), Ban Xia (*Pinellia ternata* (Thunb.) Breit), Chen Pi (*Citrus reticulata* Blanco), Fu Ling (*Poria cocos* (Schw.) Wolf), Gan Cao (*Glycyrrhiza uralensis* Fisch.), Qian Hu (*Peucedanum praeruptorum* Dunn), Jie Geng (*Platycodon grandiflorum* (Jacq.) A. DC.), Zhi Ke (*Citrus aurantium* L.), Sheng Jiang (*Zingiber officinale* Rosc.), and Da Zao (*Zizyphus jujuba* Mill.)	Cool dryness attacks the lung	15.9	3,159 (12.8%)

Mai-Men-Dong-Tang	Mai Men Dong (*Ophiopogon japonicus* Ker-Gawl.), Ban Xia (*Pinellia ternata* (Thunb.) Breit), Ren Shen (*Panax ginseng* C. A. Meyer), Gan Cao (*Glycyrrhiza uralensis* Fisch.), Da Zao (*Zizyphus jujuba* Mill.), and Geng Mi (Oryza sativa L.)	Lung and stomach Yin deficiency with deficient fire flaming upward	12.4	2,640 (10.7%)

Zhi-Sou-San	Jie Geng (*Platycodon grandiflorum* (Jacq.) A. DC.), Jing Jie (*Schizonepeta tenuifolia* (Benth.) Briq.), Zi Wan (*Aster tataricus* L. f.), Bai Qian (*Cynanchum stauntonii* (Decne.) Schltr. Ex Levl. or *Cynanchum glancescens* (Decne.) Hand.-Mazz.), Chen Pi (*Citrus reticulata* Blanco), and Gan Cao (*Glycyrrhiza uralensis* Fisch.)	Lung Qi failing to diffuse due to external pathogens	15.0	2,594 (10.5%)

**Table 2:** Single herbs (SH) frequently used for asthmatic children in Taiwan (total prescriptions: 24,650).

English name	Latin name	Indication	Average dose (gm/day)	Prevalence (%)
Zhe Bei Mu	*Fritillaria thunbergii* Miq.	Lung heat with phlegm	4.2	3,605 (14.6%)
Xing Ren	*Prunus armeniaca* L.	Constipation	3.3	2,633 (10.7%)
Jie Geng	*Platycodon grandiflorum* (Jacq.) A. DC.	Lung phlegm, pus	3.6	2,629 (10.7%)
Huang Qin	*Scutellaria baicalensis* Georgi	Lung heat, phlegm	3.7	1,991 (8.1%)
Yu Xing Cao	*Houttuynia cordata* Thunb.	Lung heat, pus	4.1	1,634 (6.6%)
Gan Cao	*Glycyrrhiza uralensis* Fisch.	Cough, smooth muscle spasm	2.1	1,413 (5.7%)
Gua Lou Zi	*Trichosanthes kirilowii* Maxim. or *Trichosanthes rosthornii* Harms.	Lung heat, phlegm, constipation	3.7	1,379 (5.6%)
Sang Bai Pi	*Morus alba* L.	Lung heat	3.3	1,314 (5.3%)
Qian Hu	*Peucedanum praeruptorum* Dunn	Lung heat, phlegm	3.7	1,176 (4.8%)
Kuan Dong Hua	*Tussilago farfara *L.	Lung phlegm, cough	3.0	1,007 (4.1%)

**Table 3:** Important drug pairs for asthmatic children (total prescriptions: 24,650).

Combination of two CHM	Prescription counts (%)
Ma-Xing-Gan-Shi-Tang with Zhe Bei Mu	871 (3.5%)
Xing Ren with Zhe Bei Mu	822 (3.3%)
Jie Geng with Zhe Bei Mu	720 (2.9%)
Ma-Xing-Gan-Shi-Tang with Jie Geng	649 (2.6%)
Ma-Xing-Gan-Shi-Tang with Huang Qin	552 (2.2%)
Huang Qin with Zhe Bei Mu	537 (2.2%)
Ma-Xing-Gan-Shi-Tang with Sang-Ju-Yin	502 (2.0%)
Ma-Xing-Gan-Shi-Tang with Sang Bai Pi	474 (1.9%)
Ma-Xing-Gan-Shi-Tang with Yu Xing Cao	474 (1.9%)
Ma-Xing-Gan-Shi-Tang with Xin-Yi-Qing-Fei-Tang	457 (1.8%)

**Table 4:** Possible pharmacological mechanisms of CHM frequently used for asthmatic children.

CHM	Active ingredients	Possible mechanisms	References
*Single herb (SH) *			
Zhe Bei Mu	Ethanol extract	Decreases serum IL-6, IL-8, and TNF-alpha and inhibits MAPK pathway	[38]
Xing Ren	Aqueous extract	Inhibits type 2 T-helper cell	[39]
Jie Geng	Aqueous extract	Decreases serum IgE, ROS scavenger	[40]
	Saponin	Decreases Syk-dependent cascades and inhibits downstream MAPK, Akt pathway	[41]
	Ethanol extract	Decreases serum IL-6, prostaglandin D_2_, leukotriene C_4_, beta-hexosaminidase, and COX-2 protein	[42]
Huang Qin	Skullcapflavone II	Bradykinin antagonist, decreases type 2 T-helper cells and increases transforming growth factor-beta 1	[43]
	Ethanol extract	Restores serum IL-8 and TNF-*α* and inhibits MAPK expression	[44]
Yu Xing Cao	Ethanol extract	Suppresses Th2 responses decreases subsequent expression of IL-4 and thymus and activation-regulated chemokine	[45]
Gan Cao	Flavonoid	Inhibits secretion of eotaxin-1 and decreases eosinophil recruitment in asthmatic airway	[46]
		Prevents eosinophil cationic protein interaction	[47]
		Suppresses memory Th2 cells, decreases serum IL-4, IL-13, and increases IFN-gamma	[48]
Sang Bai Pi	Water extract	Suppresses Th2 cell and enhances regulatory T cells	[49]
Qian Hu	(±)-Praeruptorin A	Relieves airway inflammation, airway hyperresponsiveness, and remodeling via suppressing Th2 cell	[50, 51]
Kuan Dong Hua	Water extract	Antitussive effect	[52]
	Chlorogenic acid, 3,5-dicaffeoylquinic acid, and rutin		

*Herbal formula (HF) *			
Ma-Xing-Gan-Shi-Tang (MXGST)		Antitussive effect	[21]
		Beta-2 androgenic effect	[20]
Xiao-Qing-Long-Tang (XQLT)		Relieves airway inflammation, remodeling, and immunomodulation, via downregulating IL-10, IL-13, RANTES, Eotaxin, and MCP-1	[26]
		Anti-inflammatory effect, decreasing IgE titer in bronchial lavage fluid.	[25]
		Suppresses histamine signaling	[36]
		Suppresses Th2 cell, decreasing IL-4 and IL-5, restoring IFN-gamma	[34, 35]
		Bronchial dilation, partial beta-2 androgenic effect	[53]
Mai-Men-Dong-Tang		Antitussive effect	[54]
		Bronchial dilation via beta-2 androgenic effect	[55]
